# Transient Intussusception Mimicking Acute Coronary Syndrome

**DOI:** 10.1155/2023/7324188

**Published:** 2023-10-09

**Authors:** Hiroshi Imamura, Yuichiro Kashima, Yujiro Hamano, Aoi Ogawara

**Affiliations:** Department of Emergency and Critical Care Medicine, Shinshu University, Matsumoto, Japan

## Abstract

Intussusception in adults is rare and usually associated with organic lesions. However, in the current era of computed tomography (CT), cases of idiopathic and transient intussusceptions are being increasingly diagnosed. Herein, we present a case of ileocecal intussusception with symptoms mimicking those of acute coronary syndrome. A male patient in his 80s with a history of myocardial infarction presented to the emergency department with acute onset of severe precordial and epigastric pain, cold sweating, and vomiting. Coronary angiography did not reveal any significant new lesion, while abdominal CT revealed ileocecal intussusception without bowel obstruction. The pain spontaneously subsided without any intervention, and the patient was discharged on the sixth hospital day. Cases of intussusception may go unnoticed in patients suspected of having chest pain with a normal coronary arteriogram, as idiopathic intussusception is relatively common and subsides spontaneously. Therefore, physicians should note that intussusception is one of the differential diagnoses of acute coronary syndrome.

## 1. Introduction

Adult intussusception is a rare condition and is usually associated with organic lesions, such as neoplasms and infections [1, 2]. However, with the current use of computed tomography (CT), there has been an increase in the diagnosis of idiopathic and transient intussusceptions [3–5]. Here, we present a case of ileocecal intussusception that mimicked symptoms of acute coronary syndrome. The symptoms spontaneously subsided without any intervention. It is possible that cases of intussusception may go unnoticed in patients suspected of having chest pain with a normal coronary arteriogram, as idiopathic intussusception is relatively common and subsides spontaneously.

## 2. Case History

A male patient in his 80s was transferred to the emergency department after experiencing a sudden onset of severe precordial and epigastric pain, cold sweating, and vomiting while working on the farm. Upon arrival of the emergency medical technicians, he appeared anguished and pale and complained of chest pain (8 on a 10-point scale) identical to that during his previous myocardial infarction. His vital signs were stable: pulse rate, 74 beats per min; blood pressure, 117/63 mmHg; and respiratory rate, 24 cycles per min. During transport, he vomited twice, but the pain gradually decreased.

His past medical history included posterolateral myocardial infarction a year before admission, which was treated with percutaneous coronary intervention. He had hypertension, dyslipidemia, and a history of ureterolithiasis. His medications included aspirin, prasugrel, carvedilol, amlodipine, azilsartan, and rosuvastatin. He was a former smoker and social drinker but denied illicit drug use. His family history was unremarkable.

Upon arrival, which was approximately 1 h after symptom onset, his pain subsided, and he experienced severe sweating. His vital signs were stable. His oxygen saturation was 99% on 4 L per min of oxygen via a face mask, and his temperature was 36.2°C. His lungs were clear, and his heart sound was normal. Mild tenderness of the abdomen was observed, and his physical examination was otherwise unremarkable. Electrocardiography and chest radiography revealed no abnormalities. Laboratory investigations revealed an increased white blood cell count of 12.81 × 10^9^/L, normal troponin T and C-reactive protein levels of 0.015 ng/mL and 0.01 mg/dL, respectively, and slightly elevated serum D-dimer levels of 1.2 *µ*g/ml. Transthoracic echocardiography revealed slightly decreased wall motion of the lateral left ventricular wall, similar to that observed during his previous myocardial infarction.

### 2.1. Differential Diagnosis

**Figure 1:**
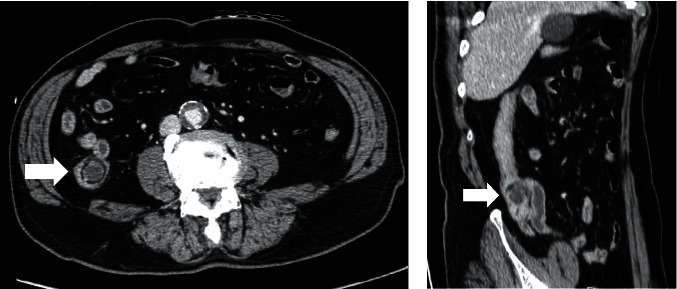
Contrast-enhanced axial (a) and coronal oblique (b) computed tomography of the abdomen shows a “bowel-in-bowel” appearance (white arrows) in the right lower quadrant. This appearance was consistent with ileocecal intussusception, and there was no evidence of proximal obstruction.

Based on his symptoms and cardiac history, acute coronary syndrome (ACS) was initially suspected. Emergent coronary angiography revealed only mild-to-moderate stenosis of the diagonal branch at the site of stent implantation. Chest and abdominal CT revealed ileocecal intussusception without bowel obstruction (Figure 1). There was no evidence of aortic dissection, pulmonary embolism, or biliary disease.

### 2.2. Outcome and Follow-Up

After admission, he was treated with conservative management and experienced no pain, vomiting, diarrhea, or hematochezia. His white blood cell count decreased to 8.37 × 10^9^/L, but his C-reactive protein levels were slightly elevated (0.48 mg/dL) the next day. There was no increase in troponin T levels. Follow-up abdominal CT on the fourth hospital day revealed the disappearance of intussusception. A colonoscopy performed on the fifth day showed no abnormal findings.

The patient was diagnosed with transient idiopathic intussusception and was discharged on the sixth hospital day. At the 5-year follow-up, the patient was in good condition with no precordial or abdominal pain.

## 3. Discussion

Adult intussusception is a rare condition, accounting for only 5% of all cases of intussusception and 1–5% of intestinal obstruction cases in adults. Adult intussusception is distinct from pediatric intussusception in various aspects. In children, it is usually primary and benign, and pneumatic or hydrostatic reduction of the intussusception is sufficient to treat the condition in 80% of cases. In contrast, almost 90% of the cases of adult intussusception are associated with organic lesions [1, 2]. The pathological lesions, such as carcinoma, polyps, Meckel's diverticulum, colonic diverticulum, strictures, or benign neoplasms, serve as the lead point. The clinical presentation of adult intussusception varies considerably. The classic pediatric presentation of acute intussusception (a triad of cramping abdominal pain, bloody diarrhea, and a palpable tender mass) is rare in adults. Nausea, vomiting, gastrointestinal bleeding, change in bowel habits, constipation, and abdominal distension are the nonspecific symptoms and signs of intussusception. Due to the significant risk of associated malignancy, radiologic decompression is not addressed in adults; therefore, 70–90% of cases of adult intussusception require surgery.

In recent years, CT has become the preferred imaging modality for the evaluation of acute abdominal pain. Evidence of intussusception on CT includes a target sign, a sausage-shaped mass with different layers of attenuation, and intraluminal fat. As the obstruction progresses, proximal dilation and distal decompression with increasing bowel wall edema and vascular compromise are seen.

In the pre-CT era, idiopathic intussusception accounted for less than 15% of adult intussusception cases. However, several recent reports have found idiopathic intussusception to account for 48–94% of cases. This discovery is likely attributed to the routine use of CT, which can identify a new subset of patients with intussusception and mild symptoms. Thus, idiopathic intussusception is more common than was previously thought.

The most common location in the gastrointestinal tract where an intussusception can occur is the junction between freely moving segments and retroperitoneally or adhesionally fixed segments. Based on their location, intussusceptions have been classified into four categories: (1) entero-enteric; (2) colic-colic; (3) ileo-colic, defined as the prolapse of the terminal ileum within the ascending colon; and (4) ileo-cecal, wherein the ileo-cecal valve is the leading point of the intussusception, as in our case.

Our patient's epigastralgia seems to represent a referred pain resulting from visceral innervation of the ileocecal region [6]. Pain arising from visceral organs is produced by stimuli that include hollow organ stretch or distension, traction on the mesentery, and organ hypoxia or ischemia. The visceral afferent fibers terminate in the superficial laminae of the spinal dorsal horn, which is also the principal site of spinal termination of the somatic nociceptive afferents. At the spinal cord level, virtually all second-order spinal dorsal horn neurons that receive a visceral input also receive convergent somatic input from the skin and/or muscles. Such viscerosomatic convergence onto second-order spinal neurons is considered the underlying structural basis for the referral of visceral sensations and pain to somatic (nonvisceral) sites. Commonly, second-order spinal neurons also receive convergent input from other visceral organs, contributing to cross-organ sensitization via visceral-visceral convergence. Cross-organ sensitization most commonly arises between organs within the thoraco-upper abdominal areas. Thus, it might be possible that our patient had pain identical to that during his previous myocardial infarction due to ileocecal intussusception. Cold sweating may have been caused by transient intestinal ischemia. This case posed a diagnostic dilemma because of the patient's prior cardiac history and symptoms mimicking those of myocardial ischemia.

Differential diagnoses of ACS include many subdiaphragmatic diseases, such as peptic ulcers, biliary disease, and pancreatitis. These subdiaphragmatic diseases are usually diagnosed based on physical findings and laboratory or imaging tests. However, to date, there have been no reports of intussusception cases presenting with symptoms mimicking those of ACS. Patients with symptoms and signs suggestive of ischemic heart disease but found to have no obstructive coronary arteries on angiography are often diagnosed with ischemia with nonobstructive coronary arteries (INOCA) [7, 8]. Pathophysiological mechanisms responsible for INOCA include epicardial coronary artery spasm or coronary microvascular dysfunction. Therefore, it might be possible that cases of intussusception may have been missed in patients who were suspected of INOCA, as idiopathic intussusception is relatively common and subsides spontaneously.

Historically, adult intussusception has been treated surgically owing to the association of pathology serving as the lead point. More recently, however, the widespread use of CT/magnetic resonance imaging has resulted in an increased frequency of the radiographic diagnosis of intussusception. Recent studies suggest that intussusceptions that lack a pathological cause of obstruction on CT are likely self-limiting and do not require surgery [3–5]. As such, retrospective studies have demonstrated successful nonoperative management in as many as 82% of cases. Despite the increase in the number of idiopathic cases of intussusception, Onkendi et al. reported that 70% of patients have a pathologic lead point, including cancer [4]. Our patient did not have either a pathological lesion or a lead point on CT, and his symptoms subsided spontaneously. Thus, he could be managed nonoperatively. Moreover, the colonoscopy showed no pathological lesions.

## 4. Conclusion

In conclusion, physicians should note that intussusception is one of the differential diagnoses of ACS.

## Data Availability

The data that support the findings of this study are available from the corresponding author upon request.
